# Rapid suspension-based screening of *Trichinella, Alaria* and *Sarcocystis* spp. in game and processed meat

**DOI:** 10.1016/j.ijppaw.2026.101223

**Published:** 2026-03-30

**Authors:** Alexandra Juhász, Gábor Majoros

**Affiliations:** a Institute of Medical Microbiology, Semmelweis University, H-1089, Budapest, Hungary; b Department of Tropical Disease Biology, Liverpool School of Tropical Medicine, Liverpool, L3 5QA, UK; c Private Scholar, István Str. 49, H-1078, Budapest, Hungary

**Keywords:** *Alaria* trematodes, Foodborne parasites, Meat inspection, *Sarcocystis* spp., *Trichinella* spp., Zoonoses

## Abstract

Foodborne zoonoses affect nearly 10% of the global population each year, with parasitic disease making up a significant proportion of these infection. Meat-borne parasites of zoonotic relevance, including *Trichinella* spp., *Sarcocystis* spp., and *Alaria alata*, can be transmitted through pork and other meats, with trichinellosis remaining a significant public health concern despite effective control in domestic pigs. This study introduces a simple, rapid suspension-based method for detecting major meat-borne parasites in fresh and processed meat. The method is intended for rapid, field-applicable detection in research or monitoring where laboratory equipment is limited. Encapsulated *Trichinella* spp. larvae and *Alaria* spp. mesocercariae were obtained from naturally infected wild boar muscle and spiked into non-infected pork, minced meat, and sausages (five capsules and five larvae per 50 g; n = 15). Samples were blended with water, filtered, stained with Nile blue or neutral red, cleared with dilute acetic acid, and examined under a stereomicroscope. All spiked *Trichinella* and *Alaria* larvae were detected in the experimentally prepared samples under controlled spiking conditions. This corresponds to an experimental recovery level of five larvae per 50 g sample. These findings reflect observations obtained under laboratory conditions and do not represent formally validated diagnostic performance parameters. The method also visualised dead or calcified *Trichinella* capsules and *Sarcocystis* cysts within muscle fibres, demonstrating its applicability for detecting multiple parasites in muscle tissue. While not intended to replace official digestion tests, this low-cost method provides a practical tool for detecting multiple meat-borne parasites in fresh and processed tissues. It can enhance field diagnostics and research in resource-limited settings, supporting food safety surveillance, public health, and studies of parasite co-infections in wild or domestic animal populations.

## Highlights

• Rapid method detects *Trichinella*, *Alaria*, and *Sarcocystis* spp. in meat.
• Method identifies live, dead, and calcified *Trichinella* larvae easily.
• Method works on raw, minced, and processed meat under field conditions.
• Enables simultaneous detection of multiple meat-borne parasites in one assay.
• Provides a practical tool for food safety monitoring in resource-limited settings.

## Graphical abstract

**Figure:**
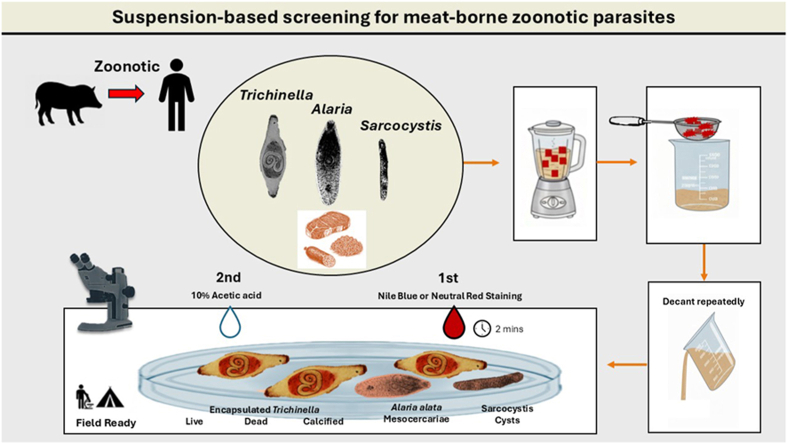

## 1 Introduction

Foodborne zoonoses, particularly those transmitted via meat, constitute a significant global public health concern, with parasitic infections contributing substantially to this burden (Kagira et al., 2009; Djurković-Djaković et al., 2013). The increasing popularity of free-range farming, consumption of game meat, and organic production systems may enhance human exposure to meat-borne parasites due to closer interactions between wildlife and livestock. Among these, *Trichinella* spp., *Alaria alata*, *Sarcocystis* spp., and *Taenia solium* are frequently detected in pork and wild game (Freeman et al., 1976; Korpysa-Dzirba et al., 2021; Klich et al., 2022; Malone et al., 2024).

*Trichinella* and *Alaria* species are of particular zoonotic relevance. Both parasites are commonly associated with the consumption of wild boar *(Sus scrofa)* meat (Ch'ung-sung, 1958; Castaño Zubieta et al., 2014; Bandino et al., 2023). Despite effective control programs reducing *Trichinella spiralis* infections in domestic pigs, the sylvatic cycle maintained by wildlife continues to pose a risk (Rostami et al., 2017; Gherman et al., 2022; Pozio, 2022). Globally, approximately 66,000 human cases were reported between 1986 and 2009 (Murrell and Pozio, 2011). The ability of *Trichinella* spp. to thrive in diverse hosts and environments complicates its control (Wakelin, 1993; Gottstein et al., 2009; Hill et al., 2010). Although severe or fatal cases are rare, human trichinellosis underscores the need for continuous surveillance and preventive measures. (Rostami et al., 2017). Similarly, *A. alata* is widespread among wild boars across Europe, and its mesocercariae have been detected in raw or undercooked game meat (Edwards et al., 2014; Korpysa-Dzirba et al., 2021). As both *Trichinella* spp. and *Alaria* spp. may co-occur in the same host, the development of diagnostic methods capable of detecting multiple meat-borne parasites is increasingly important. Moreover, wild boars may also harbour *Sarcocystis* spp., some of which are zoonotic and transmitted via undercooked meat (Dubey et al., 2016; Rosenthal, 2021; Brown et al., 2025).

Molecular methods offer high sensitivity for parasite identification (Gazzonis et al., 2019) but require laboratory infrastructure, limiting their applicability under field conditions. The standard artificial digestion technique is the gold standard for *Trichinella* spp. detection; however, it is laborious, costly, and cannot simultaneously detect other zoonotic parasites that may be present in meat (Nöckler et al., 2000; Noeckler et al., 2019). The suspension-based method described here provides a practical complementary approach for rapid on-site detection of meat-borne parasites in domestic and wild animals. Here, we present a simplified suspension-based method for the simultaneous detection of *Trichinella* spp., *Alaria* spp., and *Sarcocystis* spp. not only in raw but also in processed meat where the parasites are dead and their body are not resistant against liquefying effect of artificial digestion. This low-cost, field-applicable approach enhances food safety monitoring and parasitological surveillance in domestic and wild animals.

## 2 Materials and methods

### 2.1 Sample preparation

Muscle samples from wild boar (*Sus scrofa*) carcasses, naturally infected with *Trichinella* species, were examined by trichinoscopy (compressorium technique) to reveal presence of encapsulated *Trichinella* larvae. Encapsulated *Trichinella* larvae were manually excised together with small fragments of surrounding muscle tissue from naturally infected wild boar samples. From each wild boar muscle sample, pieces of muscle containing five *Trichinella* capsules were excised and each set of five capsules was homogenized into 50 g of *Trichinella*-negative pork. The same spiking procedure was applied to *Trichinella*-negative minced pork and to commercially produced pork sausages (50 g portions); five encapsulated *Trichinella* larvae were added per 50 g portion. Each meat type was prepared in quintuplicate (n = 5 independent 50 g replicates per matrix). Additionally, five *Alaria* spp. mesocercariae were added to each 50 g replicate of the three meat matrices (n = 5 replicates per matrix). Wild boar muscle samples naturally contained *Sarcocystis* spp. cysts, which were therefore present in the spiked test samples. These cysts were not visible in unstained compressed muscle preparations but were detected by the described suspension-based method. Pork muscle, minced meat and sausage portions were cut into ∼1 cm^3^ pieces using scissors. Samples were processed within 1 h of spiking and stored at room temperature prior to analysis. Each 50 g sample was placed in a domestic blender (Tefal Blendforce II, model BL420840, 600 W; Tefal Group, Rumilly, France) with cold tap water (approx. 200 mL) sufficient to submerge the blades and homogenized for 3–4 min at full speed to obtain a uniform suspension. The suspension was diluted with tap water to a final volume of 1 L to facilitate subsequent filtration.

### 2.2 Filtration and sedimentation

The suspension was passed through a 1.0–2.0 mm mesh pasta strainer into a 1 L plastic bucket. The blender container was rinsed with cold water, and the washings were filtered through the strainer into the bucket. Tissue debris retained in the strainer or on the blender blades (visible connective tissue and fat) was discarded. The suspension was diluted with additional cold water and left to settle for 7–8 min at room temperature (∼20–22 °C). The supernatant was carefully decanted, and fresh water was added to repeat the sedimentation process. The washing and sedimentation cycle was repeated 2–3X until the supernatant was clear of fat droplets and fine, light-pink muscle fragments were observed at the bottom.

### 2.3 Staining and microscopy

A portion (∼5 mL) of the sediment were transferred to Petri dishes or white porcelain plates. Nile blue or neutral red dye solution (0.5–1 mL for 1% solution or 3–4 mL for 0.1%) was added to the sediment and mixed until the suspension was lightly coloured. The dye adhered to the solid particles with the surrounding water becoming nearly colourless within 2 min. To enhance contrast and visibility, 10% acetic acid was added dropwise until the background cleared (typically 8-10 drops). The acid cleared the background by removing dye from fragmented muscle fibres without blenching the more compact structures, such as the *Trichinella* capsules and other parasites covered with a membranous layer. Samples were examined using a stereo microscope at 20–30 × magnification (binocular, 10 × oculars, objective 0.5–5 × ) with top illumination. *Trichinella* capsules, if present, were identified as spindle-shaped structures that stained blue or red, depending on the dye used. Nile blue and neutral red were selected due to their strong affinity for compact structures containing numerous nuclei or necrotic material. This ensures high visibility under a stereo microscope, even after acetic acid treatment. A stereo microscope with at least 20 × magnification and top illumination is sufficient for examination. An optimal setup includes a binocular stereo microscope with 10 × oculars and objective lenses ranging from 0.5 × to 5 × magnification. The characteristic morphology of larvae inside the capsule or presence of other parasites allowed for further verification under higher magnification if required.

## 3 Results

The suspension-based method was evaluated on 15 laboratory-prepared samples consisting of pork muscle, minced meat, and sausage, each spiked with five encapsulated *Trichinella* spp. larvae and five *Alaria* spp. mesocercariae per 50 g of non-infected meat (n = 15). Unknown *Sarcocystis* spp. cysts naturally present in the same tissues were also included for visual assessment (Table 1). All artificially spiked *Trichinella* and *Alaria* larvae were detected in all experimentally prepared samples. This corresponds to the recovery of five larvae per 50 g portion under controlled laboratory spiking conditions. No formal limit-of-detection assessment was performed. The technique also enabled the detection of calcified and necrotic *Trichinella* capsules, as well as *Sarcocystis* cysts, which were not visualised by compression or digestion methods.

**Table 1:** *Detection of spiked Trichinella spp. Larvae, Alaria alata mesocercariae and Sarcocystis* spp. cysts *in different meat sample types using the suspension-based method*.

Sample type	No. of spiked *Trichinella* capsules and *Alaria alata* mesocercariae	Sample weight (g)	Suspension result for *Trichinella* capsules and *Alaria alata* mesocercariae	Comment
Pork muscle chunks	5-5	50	Positive (5/5)	*6 Sarcocystis s*pp. cyst in the sample
Minced meat	5-5	50	Positive (5/5)	*7 Sarcocystis s*pp. cyst in the sample
Sausage	5-5	50	Positive (5/5)	*9 Sarcocystis s*pp. cyst in the sample

**Total tested**	**15**–**15**	**150**	**15/15 positive**	**22 *Sarcocystis s*pp. cyst**

**Fig. 1:**
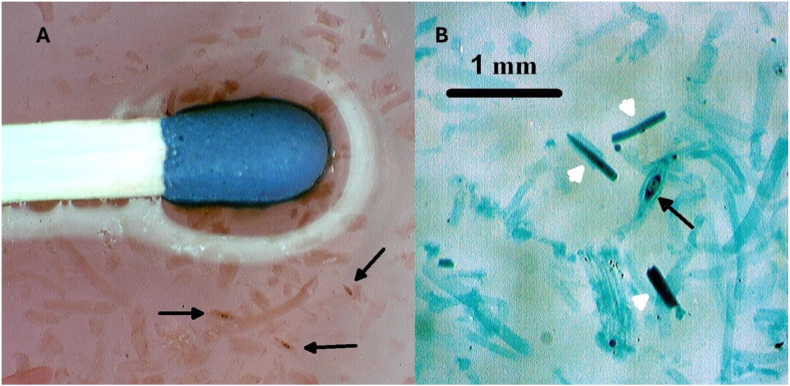
Size comparison and visualization of *Trichinella* cysts and larvae A) Size of *Trichinella* cysts in a Petri dish compared to a matchstick, stained with neutral red (size of capsules: ∼500 μm). B) *Trichinella* capsule with larvae (black arrow) and *Sarcocystis* spp. cysts (white arrow) in Petri-dishes from wild boar at 50X magnification stained with Nile blue.

**Fig. 2:**
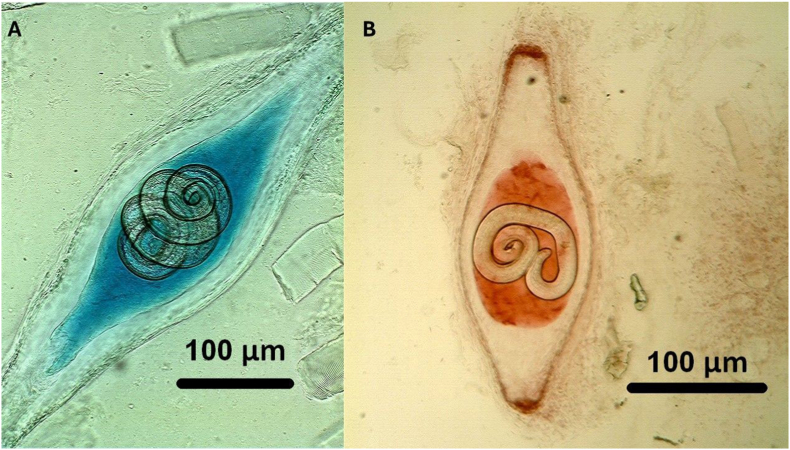
*Trichinella* spp. and additional parasites detected using differential staining. *Trichinella* capsule with larva at 700X magnification, A) stained with Nile blue, B) stained with neutral red at 700X magnification.

Distinct spindle-shaped *Trichinella* capsules, containing intact larva were clearly visible under stereomicroscopy, retaining intense blue or red coloration depending on the dye used (*Nile blue* or *neutral red*). Due to their higher density, the stained capsules settled quicker than the fragmented muscle fibres at the bottom of the suspension after decantation, even following mild acetic acid treatment (Fig. 1, Fig. 2), allowing clear differentiation from surrounding tissue debris. Metabolic residues around the larvae, primarily phospholipids and degraded material, also retained the dyes, enhancing visual contrast.

**Fig. 3:**
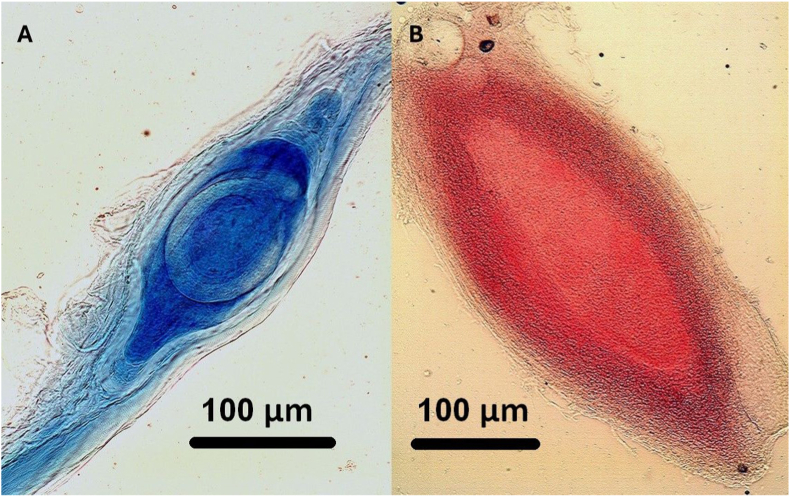
Detection of dead or calcified *Trichinella* larvae using staining techniques. A) Capsule with dead, necrotised larva stained with Nile blue. B) Fully necrotised capsule without recognizable larva stained with neutral red (Magnifications as same as in case of Fig. 2).

Capsules lacking live larvae exhibited stable and distinct staining, allowing recognition of calcified or dead *Trichinella* larvae (Fig. 3). Although staining intensity gradually faded with prolonged acid exposure, it remained adequate for reliable microscopic evaluation throughout the observation period.

The method performed consistently across all sample types, including fatty matrices such as minced meat and sausage, without reduction in parasite detectability. Occasionally, inorganic particles such as lime sediment were observed but could be readily distinguished, as they did not retain dye or they have irregular shape. The complete test procedure, including sample preparation and equipment cleaning, required approximately 1 h per operator.

**Fig. 4:**
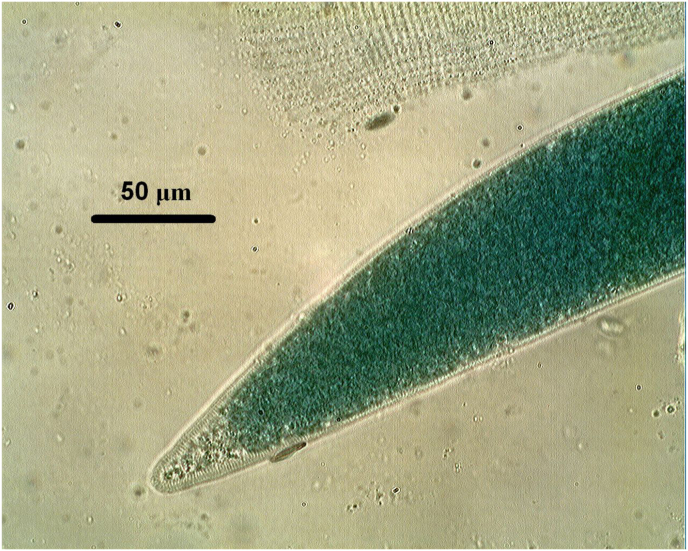
*Sarcocystis* spp. cyst released from muscle fibre, providing similar staining by Nile blue and size to *Trichinella* capsules, aiding diagnostic accuracy. (1000x magnification).

**Fig. 5:**
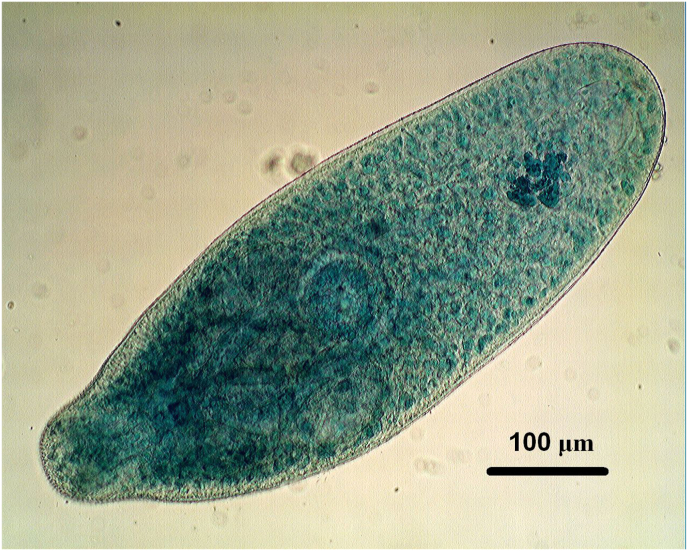
Nile blue stained *Alaria* larva in muscle tissue enhancing diagnostic scope by using the suspension method. (200x magnification).

In addition to *Trichinella* and *Alaria*, the suspension method enabled the simultaneous detection of *Sarcocystis* spp. cysts in the same muscle samples (Fig. 4) and identified co-occurring zoonotic parasites such as *Alaria alata* mesocercariae within the same tissues (Fig. 5). Due to the lack of test samples containing such parasites, we did not have the opportunity to more precisely determine the sensitivity of the proposed method, but it was clearly demonstrated that several parasites can be detected in the same way with this procedure.

## 4 Discussion

### 4.1 Principle of the test method

The average density of parasites in meat is always slightly higher than the density of the surrounding muscle tissue, because they contain more cells (nuclei) per unit volume than muscle tissue. Therefore, they settle faster in suspended tissue debris than muscle fibers.

The dyes used in the method stain parasites more intensely than muscle tissue containing few nuclei, because due to the many nuclei, they contain more phospholipids and, after their destruction, more fatty acids than intact muscle. Therefore, neutral red and Nile blue, which have long been used for histological examinations (Romeis, 1948), are also suitable for selective staining of parasites. The dye can be used in a similar way to make dead parasites visible and to recognize tumor cells (Lin et al., 1991). Acetic acid penetrates muscle fibers faster than parasites, so the latter fade more slowly under the influence of the acid.

### 4.2 Advantages and disadvantages of the simplified suspension method for meat-borne parasites examination

The suspension method proved particularly useful under field conditions and in rural areas where access to accredited laboratories is limited, enabling on-site inspections with minimal resources. Artificial digestion remains the internationally accepted reference method for *Trichinella* detection (Forbes and Gajadhar, 1999). The suspension-based approach is not intended to replace regulated inspection protocols but may serve as a complementary exploratory tool under field conditions. Unlike digestion, which dissolves non-viable larvae and capsules, the suspension technique preserves both viable and non-viable structures, which may provide additional epidemiological information. However, formal comparative validation studies are required to define its diagnostic performance relative to established methods. By detecting calcified and necrotic larvae in addition to live ones, it complements standard artificial digestion and trichinoscopy, providing additional epidemiological information that might otherwise be overlooked. Compared with trichinoscopy, the method allows processing of larger sample volumes while allowing examination of larger sample volumes under field conditions. These characteristics make it a valuable complementary tool for improving food safety surveillance, especially in resource-limited settings.

The suspension method efficiently separated interfering components such as fat and connective tissue. Fat droplets floated and were removed during decantation, whereas connective tissue fibres adhered to the blender blades or were retained by the strainer. Residual fat exhibited a characteristic red-brown coloration after acetic acid exposure, allowing easy distinction from parasite structures, even in high-fat matrices such as minced meat or sausages.

Those *Trichinella* larvae that may have escaped from their capsules and those which have not yet settled inside a muscle cell are paler in colour, but if they are alive, they can be easily recognised by their movement. Unfortunately, if these larvae are no longer alive and thus immobile during the test, they are less conspicuous than intact *Trichinella* capsules. (Live, bare larvae from raw tissue can be identified better using the digestion method.) By the suggested method, the distinctive appearance and coloration of the capsules make the presence of larvae easily recognizable.

The method also remained applicable to dried meat samples that were subsequently rehydrated, although capsules occasionally adhered to each other, resulting in weaker staining but still allowing visual identification. In marinated meat treated with mild acid or 10% vinegar, capsules often disintegrated, leaving only faintly stained, dead larvae, which can be recognised with experience. Despite these limitations, the suspension approach seems suitable for detecting *Trichinella*, *Alaria* and *Sarcocystis* spp*.* in various meat types, including dried products such as sausages and hams where parasites are typically non-viable.

In terms of specificity, the method is particularly relevant in European regions where encapsulated *Trichinella* species and other food-borne zoonotic parasites are endemic (Pozio, 2019). Encapsulated larvae are more easily differentiated from other nematode larvae than free forms, which may resemble species of unrelated taxa. Based on the present spiking experiments (five larvae per 50 g sample), the method demonstrated successful recovery under controlled laboratory conditions. However, formal sensitivity, specificity, and limit-of-detection parameters remain to be determined in future validation studies. Future studies should focus on field validation and quantitative comparison with established digestion assays to fully define sensitivity and specificity parameters.

Compared with traditional trichinoscopy under light microscopy (Kohler and Ruitenberg, 1974), the suspension-based method may facilitate improved visualization in dense or processed meat matrices; however, direct comparative validation studies are required. Although additional steps such as spreading the suspension on slides or using a compressorium can enhance visibility, they may increase examination time.

The suspension method does not dissolve tissues, so it can also detect parasites that are dissolved by enzymes. This is beneficial in cases where domestic or wild boars harbour other zoonotic food-borne parasites, such as *Sarcocystis* cysts (Helman et al., 2023). *Sarcocystis* does not produce visible lesions at slaughter (Rubiola et al., 2024), but its frequent occurrence can serve as an internal procedural control during application of suspension method. These protozoan cysts can be useful in allowing examiners to calibrate visual recognition of parasitic structures due to their high frequency and similar behaviour to *Trichinella* cysts; thereby improving diagnostic accuracy (Fig. 4). In addition, the method also detects *Alaria alata* mesocercariae that may co-occur with *Trichinella* larvae in the same tissues, providing further diagnostic information (Fig. 5).

### 4.3 Potential limitations in the muscle suspension examination technique

As with any diagnostic approach, the reliability of the suspension method depends on careful execution. Excessive tissue density in the suspension or uneven distribution in the Petri dish can obscure parasites, while incomplete decantation may leave floating debris that interferes with staining. Proper decantation and clearing are therefore essential before dye application.

For aged or processed meat samples such as smoked ham or long-frozen tissue, staining intensity may decrease due to reduced dye diffusion. Adjusting staining duration or dye concentration can compensate for this effect. Because *Trichinella* capsule density varies, parasites are often concentrated near the bottom of the suspension; thus, examining multiple Petri dishes and focusing on the sedimented layer is recommended. Faint staining can be enhanced by adding additional dye, whereas overstaining can be corrected by repeated acetic acid treatment. To avoid bubble formation, stagnant water should be used rather than freshly drawn tap water.

### 4.4 Some arguments for using the muscle suspension-based method

In slaughterhouse inspections, high diagnostic sensitivity is required because pooled samples from multiple animals must be tested rapidly. However, when individual animals are examined, *Trichinella* larvae can often be detected with less sensitive yet simpler methods if larger muscle portions are taken from predilection sites. This increases the probability of larval detection even without sophisticated laboratory infrastructure.

While disease severity is generally associated with infectious dose, even low-level infections are epidemiologically relevant. Therefore, any field-applicable screening method should be interpreted with caution and does not replace regulated inspection protocols. (Dupouy-Camet, 2000; Murrell and Pozio, 2011; Malone et al., 2024).

The suspension-based method is particularly useful for rapid screening of individual samples when the origin or prior laboratory testing of the meat is uncertain. Its simplicity, speed, and low cost make it suitable for field veterinarians and private practitioners. Although artificial digestion remains the gold standard for routine meat inspection, the suspension method offers a practical, on-site alternative for identifying *Trichinella*, *Alaria*, and *Sarcocystis* spp. Its minimal equipment requirements and cost-effectiveness make it an attractive complementary tool for researchers, inspectors, and field diagnosticians**.** This quick method is also very useful if you want to demonstrate parasites in meat to students during practical training. When integrated into local surveillance programmes, it can support regulatory efforts by facilitating individual sample testing in areas lacking accredited laboratory facilities.

Overall, the suspension-based method represents a practical exploratory tool for field use, with potential complementary value once validated under controlled field conditions. It can be assumed that the method can also detect other parasites in meat (e.g. *Toxoplasma* cyst, cysticerci, *Ancylostoma, Strongyloides* larvae etc.) that we have not had the opportunity to examine.

## 5 Conclusion

This study presents a simplified suspension method as a practical and cost-effective alternative to traditional meat-borne parasites detection techniques where accredited laboratory access is unavailable. The method's versatility across different meat matrices and ease of use make it suitable for both routine inspections and emergency assessments, particularly in resource-limited or research fieldwork settings. Its ability to detect live, dead, and calcified *Trichinella* larvae, *Alaria alata* mesocercariae and *Sarcocystis* across diverse meat conditions underscores its potential to complement existing diagnostic tools. While the method does not replace the gold standard of artificial digestion mandated for public safety, it provides a valuable solution for individual meat samples, especially in scenarios where access to specialized equipment is limited. This aligns with broader goals of enhancing food safety and controlling zoonotic diseases. It bridges the gap between classical trichinoscopy and enzymatic digestion by combining simplicity with improved visualization. Future research should focus on large-scale validation under field conditions, quantitative performance comparison with digestion assays, and assessment of its integration into national food safety surveillance systems.

## CRediT authorship contribution statement

**Alexandra Juhász:** Writing – original draft, Visualization, Resources, Investigation, Formal analysis, Data curation, Conceptualization. **Gábor Majoros:** Writing – review & editing, Visualization, Validation, Supervision, Methodology, Investigation, Formal analysis, Data curation, Conceptualization.

## Ethics declarations

Ethics approval for this study was not required for using animals killed as a part of routine hunting management control program. No animal was sacrificed for this study. The wild boar sample used for analysis here was submitted for diagnostic purposes to the Department of Parasitology and Zoology, University of Veterinary Medicine.

## Declaration statement

The authors declare that they have no known competing financial interests or personal relationships that could have appeared to influence the work reported in this paper.
